# *Sporothrix* Species Causing Outbreaks in Animals and Humans Driven by Animal–Animal Transmission

**DOI:** 10.1371/journal.ppat.1005638

**Published:** 2016-07-14

**Authors:** Anderson Messias Rodrigues, G. Sybren de Hoog, Zoilo Pires de Camargo

**Affiliations:** 1 Cell Biology Division, Department of Microbiology, Immunology and Parasitology, Federal University of São Paulo (UNIFESP), São Paulo, São Paulo, Brazil; 2 Centraalbureau voor Schimmelcultures, KNAW Fungal Biodiversity Centre, Utrecht, The Netherlands; Geisel School of Medicine at Dartmouth, UNITED STATES

## The Past and Present of *Sporothrix*

The fungal genus *Sporothrix* (order Ophiostomatales) comprises a group of thermodimorphic pathogens that cause skin infections in humans and other mammals. Human sporotrichosis was first described in the Mid-Atlantic United States in 1898 by Schenck [1], followed shortly by reported animal infections [2]. Sporotrichosis occurs worldwide, with hyperendemic areas in Brazil, China, and South Africa [3,4]. Clinical sporotrichosis in mammals results from two major infection routes: animal transmission and plant origin. Both routes involve trauma to cutaneous and subcutaneous tissues to introduce *Sporothrix* propagules into the skin. Cutaneous lesions develop at the inoculation site, and local dissemination occurs through the lymphatics during the first two to three weeks of infection [5]. Infections transmitted via either animal or plant vector often escalate to outbreaks or epidemics.

**Fig 1:**
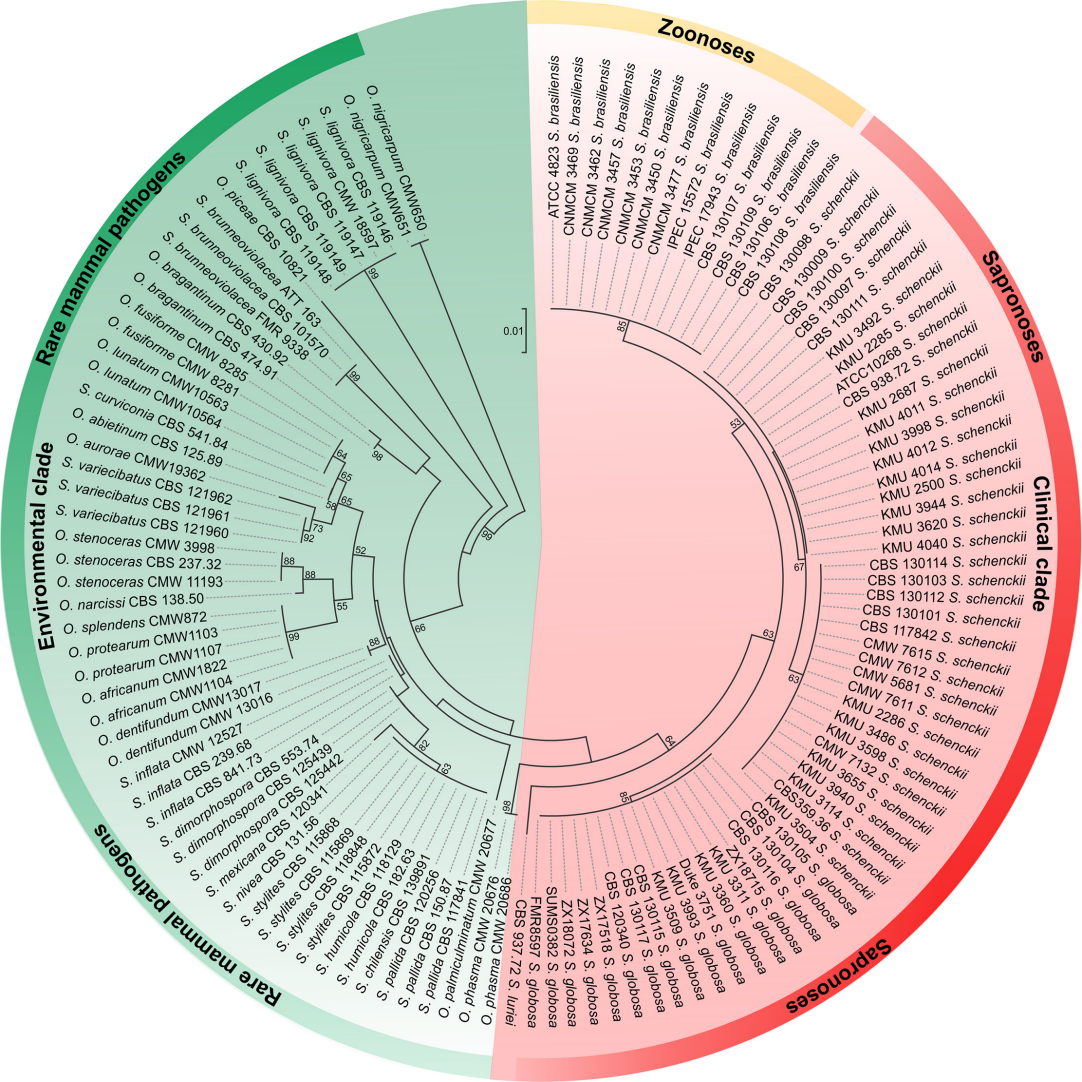
Phylogenetic analyses correlate with ecological behavior of *Sporothrix* species. Comparing the clades over the entire tree reveals a consistent decrease of bark beetle and soil association outside *Ophiostoma*, with a concomitant increase of vertebrate infectivity in *S*. *brasiliensis*, *S*. *schenckii*, and *S*. *globosa*. Phylogenetic tree generated by neighbor-joining analysis using partial nucleotide sequences of the rDNA operon (ITS1+5.8s+ITS2). Bootstrap value (1,000 replicates) was added to respective branches. Each species is indicated at its respective position in the phylogenetic tree. Bar = total nucleotide differences between taxa.

Over the last decade, molecular phylogeny has revolutionized the taxonomy of pathogenic *Sporothrix* species [6], altering our perceptions regarding epidemiology, host-association, virulence, and drug susceptibility [7–10]. The classical agent *Sporothrix schenckii* now comprises several molecular siblings nested in a clinical clade with *S*. *brasiliensis*, *S*. *globosa*, and *S*. *luriei* (Fig 1) [6]. *S*. *brasiliensis* is related to atypical and more severe clinical manifestations [11]. For decades, feline sporotrichosis in Brazil appeared only as sporadic, self-limiting clusters. However, the current outbreak of feline sporotrichosis because of *S*. *brasiliensis* in South and Southeast Brazil has risen to epidemic status, creating a public health emergency of international concern because of the potential of zoonotic transmission [9,12–15].

## Diverse Ecologies and Strategies of *Sporothrix*

Our knowledge of the natural ecology of Ophiostomatales derives from studies including basal lineages of *Sporothrix* and *Ophiostoma*, which occupy many niches (plant, soil, and bark beetles) and usually lack pathogenicity for mammals. Despite the close phylogenetic relationship with members of Ophiostomatales (Fig 1), the ecology of human-pathogenic *Sporothrix* remains enigmatic [3,4,16], particularly regarding the factors driving population dynamics and distribution ranges in nature. Species tend to appear in the form of outbreaks, which depend on specific and rare conditions in decaying plant material [4,17].

Genomic comparisons of *S*. *brasiliensis* and *S*. *schenckii* have elucidated the emergence of mammal pathogenicity in the Ophiostomatales. *Sporothrix* undergoes morphological transition in response to temperature, developing as filamentous hyphae during its saprophytic stage at 25°C or as yeast in host tissue at 37°C. Comparisons with other dimorphic fungi—including *Paracoccidioides brasiliensis*, *Histoplasma capsulatum*, *Blastomyces dermatitidis*, and *Talaromyces marneffei*—reveal multiple complex adaptive evolutionary strategies and that dimorphism in *Sporothrix* represents convergent evolution [18], shared with distant human pathogens in Onygenales and Eurotiales. This emphasizes the importance of this morphological adaptation to infection. Indeed, the global efficiency of mycelium-to-yeast transition in *Sporothrix* varies among clades that exhibit different pathogenic behaviors. For example, *S*. *brasiliensis* shows successful development of numerous yeast-like budding cells, while impaired morphological switching is seen among true environmental entities, such as *S*. *chilensis* and *S*. *mexicana*. This phenomenon could be correlated with attenuated virulence of *Sporothrix* in the environmental clade [10,19,20]. Moreover, comparative genomics has enabled identification of a contraction of plant-degrading enzymes in *S*. *brasiliensis* and *S*. *schenckii*, which has been interpreted as adaptation from plants to animals [18]. This may further support the previous epidemiological perception of *Sporothrix* making a host jump from plant to animal transmission [9].

## Transmission Types in *Sporothrix*

**Fig 2:**
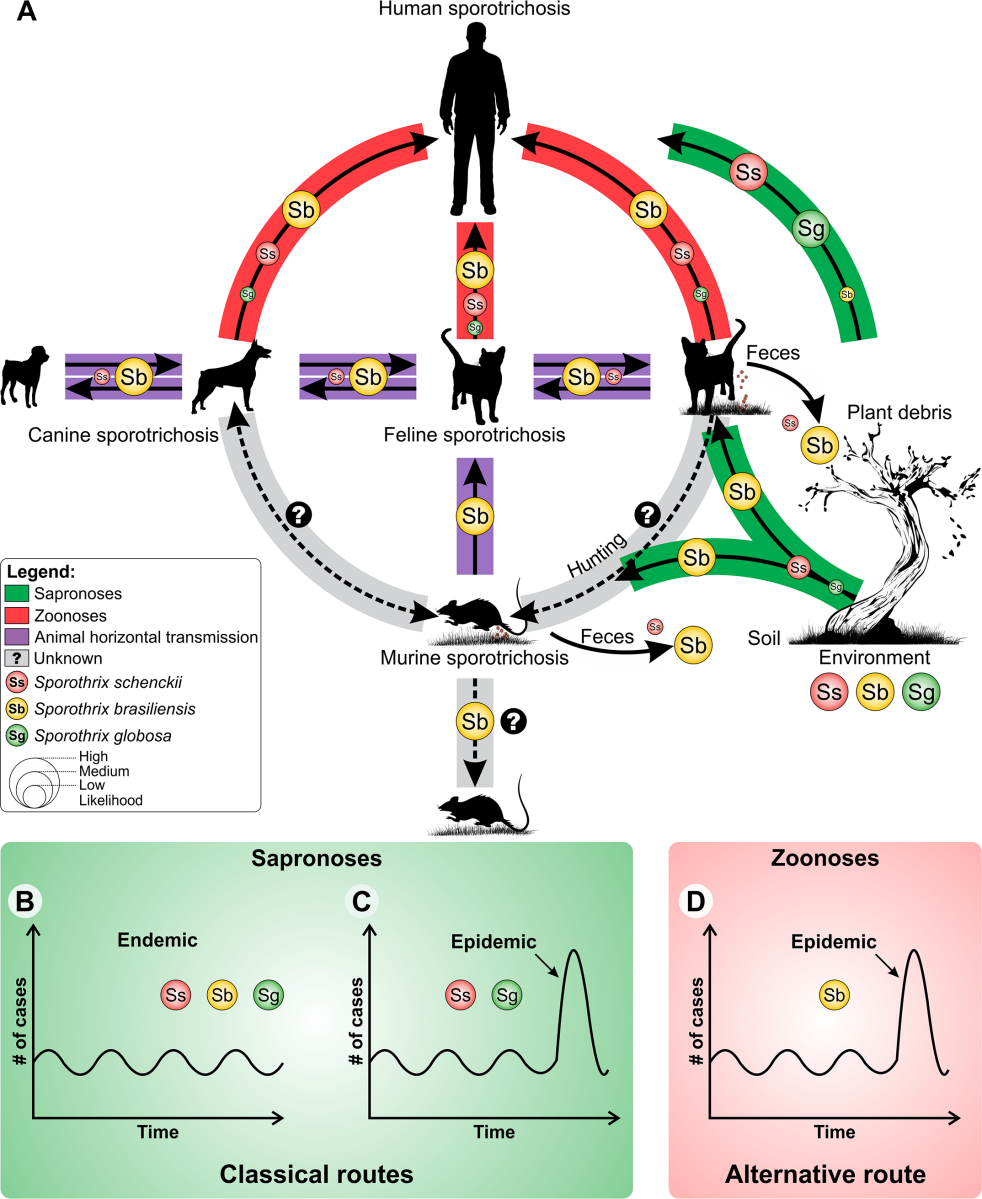
Transmission routes in human and animal sporotrichosis. The transmissibility between different species of clinical interest is explored based on epidemiological data. **(A)** *Sporothrix brasiliensis* is associated with large epizooties during animal horizontal transmission (purple route). This is not an exclusive host association, since *S*. *schenckii* may also infect cats but with lower frequency. Cat-borne sporotrichosis can be transmitted to humans (zoonoses) via deep scratching and biting, through which high loads of yeast cells are inoculated into to host tissue (red route). The threat of cross-species pathogen transmission (purple and red routes) poses the risk of a massive epidemic for humans in highly endemic areas. Note that *Sporothrix schenckii* and *Sporothrix globosa* cause large sapronoses (green route), while *S*. *brasiliensis* is less frequent during sapronoses. The size of the species’ circumference is proportional to the likelihood of involvement (high, medium, or low) in each transmission route. **(B)** In the sapronotic route (classical pathway), the presence of the etiologic agents of sporotrichosis in nature can lead to an endemic profile, with fluctuation in the number of transmissions. However, the infections remain close to the baseline over time. **(C)** Highly specific conditions must be met to promote pathogen expansion in plant debris (see [4]). **(D)** In the alternative route, feline-borne transmission via deep scratching is highly effective during animal horizontal transmission and during zoonotic transmission, placing a larger number of individuals at risk of acquiring sporotrichosis.

*S*. *schenckii* and *S*. *globosa* are cosmopolitan pathogens that generally follow an environmental transmission route via traumatic inoculation of contaminated plant debris (Fig 2A, green route). For over a century, this route has affected specific occupational populations, including agricultural workers and gardeners (Fig 2), and was termed “rose breeders’ disease.” At the other extreme, the highly virulent clonal offshoot *S*. *brasiliensis* is associated with animal infections and zoonotic transmission through deep scratches and bites from infected cats [14]. The host jump of *Sporothrix* from plant to animal transmission [9] is an important feature among the Ophiostomatales (Fig 2A, purple and red routes), distinguishing cat-transmitted sporotrichosis as an occupation-independent disease.

Numerous and large outbreaks are described in the literature [4,9,12,15,17,21]. *S*. *brasiliensis* accounts for the majority of infections via felines, with a clonal population structure observed during outbreaks. Strains with identical genotypes are found at small geographic distances [9,13]. A slow dispersal vector is likely at work. Since domestic felines are relatively sedentary, the zoonosis is expected to show slow geographical expansion (Fig 2A, purple route) [9,12]. *S*. *globosa* also shows low degrees of variation, suggesting clonality [4,6], but a major difference with *S*. *brasiliensis* is observed in that identical genotypes are repeatedly found at large geographic distances, with identical isolates originating from Colombia, China, and Brazil [4]. A rapid vector of dispersal may be responsible for the transoceanic spread of *S*. *globosa*. Given the large distances between strains, airborne dissemination via plant debris dust seems likely. Cats rarely play a role in sapronoses by *S*. *globosa* [21]. Conversely, plants are never observed as sources of infection by *S*. *brasiliensis*, and felines are responsible for continued animal–animal transmission during epizooties and zoonoses in South and Southeast Brazil (Fig 2A). In this regard, *S*. *schenckii* seems ecologically intermediate, and genetic recombination among different genotypes may have contributed to the evolution of diversity in the pathogenic clade (Fig 2A) [9].

## Drivers of *S*. *brasiliensis* Infection: Letting the Cat out of the Bag

Understanding the emergence of new and old fungal agents is critical for promoting effective public health policies [22]. Host shifts generally result from recent pathogen introduction into a susceptible host population [23–25]. Felines present a broad spectrum of clinical sporotrichosis, ranging from single lesions to fatal systemic forms [14,26]. The disease is easily transmitted, with cat-to-cat and cat-to-human transmissions occurring via deep scratching and biting that inoculates high loads of *Sporothrix* (Fig 2A). Phylogenetic data support a recent habitat shift in *Sporothrix* from plant to cat, which apparently occurred in southeastern Brazil and is supporting *S*. *brasiliensis* emergence [9]. The cat-borne, highly pathogenic *S*. *brasiliensis* depends on its feline host for its epidemic emergence [9]. Feline sporotrichosis emergence in areas where the number of cases has remained near baseline for long periods underlines the threat of cross-species pathogen transmission (Fig 2D) [9,12]. Cats are the main vectors of *S*. *brasiliensis* transmission to humans in Brazil, but the roles of other mammals (e.g., rats) should also be investigated during *S*. *brasiliensis* epizooties. Lutz & Splendore isolated pathogenic specimens from naturally infected rats [2], confirming that *Rattus norvegicus* can develop sporotrichosis. Several reports have proposed other mammals as potential carriers of *Sporothrix* propagules, emphasizing their importance in sporotrichosis transmission, including armadillos, bats, dogs, and squirrels as well as invertebrates such as mosquitoes, ants, and spiders [4].

The epicenter of the long-lasting outbreak of cat-transmitted sporotrichosis is the metropolitan region of Rio de Janeiro (Brazil), where more than 4,000 human and 4,000 feline cases were diagnosed at Fundação Oswaldo Cruz between 1998 and 2012 [14]. Similar epidemics are occurring in Rio Grande do Sul and São Paulo (Brazil), which present high prevalences of *S*. *brasiliensis* infections [12,13,15]. Urban areas with high feline population densities seem to be important drivers of epizooties because of *S*. *brasiliensis*. Outside these areas, classical transmission types prevail, with subjects mainly infected via accidental traumatic inoculation, mostly while manipulating contaminated plant material [9]. Globally, *S*. *schenckii* is the major etiological agent transmitted via the classical route [9], although *S*. *globosa* is preponderant in East Asia [4]. In endemic areas of feline sporotrichosis, early outbreak episodes are followed by massive continued transmission, suggesting negligence of the disease.

Compared to the alternative route (i.e., via the feline host, as in *S*. *brasiliensis*; see Fig 2D), the classic infection route is expected to be less effective, leading to scattered sporotrichosis cases in specific occupational groups (Fig 2B). However, large outbreaks have occurred by the classical route (Fig 2C). Large sapronoses reported from France [27], the US, [17], South Africa [28], and China [21] indicate that highly specific conditions are required to promote pathogen expansion in plant debris [4]. In the alternative feline transmission route, deep scratching is highly effective, placing larger numbers of individuals at risk of acquiring sporotrichosis (Fig 2D) [9,12,14].

## Strategies for Disease Containment

In general, zoonotic pathogens are twice as likely to be associated with emerging diseases than nonzoonotic pathogens [29]. Here, we face a novel pathogen that emerged within a highly susceptible feline population and achieved efficient and massive transmission to humans [12–14]. Moreover, *S*. *brasiliensis* virulence is higher than that of other *Sporothrix*. Epidemics driven by different transmission routes and agents with deviating virulence [7,19] and differential susceptibility to antifungals [8] necessitate *Sporothrix* diagnosis and identification for guiding public health policies and adjusting antifungal therapy [5,26,30]. Currently, two rapid assays are available to detect *Sporothrix* DNA directly from lesions and identify the pathogen with high sensitivity and specificity: rolling circle amplification [31] and species-specific PCR [30]. Likewise, serological assays, such as ELISA and immunoblot based on antigen preparations from *Sporothrix*, can detect antibodies to 3-carboxymuconate cyclase (gp60 and gp70) and may aid feline and human diagnosis [20,26] and patient follow-up [5]. Improvements in early diagnosis and surveillance systems may facilitate rapid identification and control of future outbreaks among cats and humans. Tackling an outbreak via the classical route (sapronoses) requires removal of foci in nature. It is much more difficult to control the alternative route of transmission via felines [14]. Strategies are needed to educate the population about cat maintenance, *Sporothrix* transmission, and animal sterilization, to improve early diagnosis, treatment, and prophylaxis, and to develop campaigns to prevent random abandoning of diseased animals and cadavers.

## References

[1] Schenck BR. On refractory subcutaneous abscesses caused by a fungus possibly related to the *Sporotricha* . Bull Johns Hopkins Hosp. 1898;9:286–90.

[2] Lutz A, Splendore A. [On a mycosis observed in men and mice: Contribution to the knowledge of the so-called sporotrichosis]. Revista Médica de São Paulo. 1907;21:443–50.

[3] Zhou X, Rodrigues AM, Feng P, Hoog GS. Global ITS diversity in the *Sporothrix schenckii* complex. Fungal Divers. 2014;66(1):153–65. 10.1007/s13225-013-0220-2 .

[4] Zhang Y, Hagen F, Stielow B, Rodrigues AM, Samerpitak K, Zhou X, et al Phylogeography and evolutionary patterns in *Sporothrix* spanning more than 14,000 human and animal case reports. Persoonia. 2015;35:1–20. 10.3767/003158515x687416 26823625 PMC4713101

[5] Orofino-Costa R, de Macedo PM, Bernardes-Engemann AR. Hyperendemia of sporotrichosis in the Brazilian Southeast: Learning from clinics and therapeutics. Curr Fungal Infect Rep. 2015;9(4):220–8. 10.1007/s12281-015-0235-0

[6] Marimon R, Cano J, Gené J, Sutton DA, Kawasaki M, Guarro J. *Sporothrix brasiliensis*, *S*. *globosa*, and *S*. *mexicana*, three new *Sporothrix* species of clinical interest. J Clin Microbiol. 2007;45(10):3198–206. 10.1128/JCM.00808-07 17687013 PMC2045377

[7] Fernandes GF, dos Santos PO, Rodrigues AM, Sasaki AA, Burger E, de Camargo ZP. Characterization of virulence profile, protein secretion and immunogenicity of different *Sporothrix schenckii sensu stricto* isolates compared with *S*. *globosa* and *S*. *brasiliensis* species. Virulence. 2013;4(3):241–9. 10.4161/viru.23112 . 23324498 PMC3711982

[8] Rodrigues AM, de Hoog GS, de Cassia Pires D, Brihante RSN, da Costa Sidrim JJ, Gadelha MF, et al Genetic diversity and antifungal susceptibility profiles in causative agents of sporotrichosis. BMC Infect Dis. 2014;14(1):219 10.1186/1471-2334-14-219 . 24755107 PMC4021050

[9] Rodrigues AM, de Hoog GS, Zhang Y, Camargo ZP. Emerging sporotrichosis is driven by clonal and recombinant *Sporothrix* species. Emerg Microbes Infect. 2014;3(5):e32 10.1038/emi.2014.33 26038739 PMC4051365

[10] Rodrigues AM, Cruz Choappa R, Fernandes GF, De Hoog GS, Camargo ZP. *Sporothrix chilensis* sp. nov. (Ascomycota: Ophiostomatales), a soil-borne agent of human sporotrichosis with mild-pathogenic potential to mammals. Fungal Biol. 2016;120(2):246–64. 10.1016/j.funbio.2015.05.006 . 26781380

[11] Almeida-Paes R, de Oliveira MM, Freitas DF, do Valle AC, Zancope-Oliveira RM, Gutierrez-Galhardo MC. Sporotrichosis in Rio de Janeiro, Brazil: *Sporothrix brasiliensis* is associated with atypical clinical presentations. PLoS Negl Trop Dis. 2014;8(9):e3094 10.1371/journal.pntd.0003094 25233227 PMC4169245

[12] Rodrigues AM, de Melo Teixeira M, de Hoog GS, Schubach TMP, Pereira SA, Fernandes GF, et al Phylogenetic analysis reveals a high prevalence of *Sporothrix brasiliensis* in feline sporotrichosis outbreaks. PLoS Negl Trop Dis. 2013;7(6):e2281 10.1371/journal.pntd.0002281 23818999 PMC3688539

[13] Montenegro H, Rodrigues AM, Galvão Dias MA, da Silva EA, Bernardi F, Camargo ZP. Feline sporotrichosis due to *Sporothrix brasiliensis*: an emerging animal infection in São Paulo, Brazil. BMC Vet Res. 2014;10(1):269 10.1186/s12917-014-0269-5 25407096 PMC4244058

[14] Gremião ID, Menezes RC, Schubach TM, Figueiredo AB, Cavalcanti MC, Pereira SA. Feline sporotrichosis: epidemiological and clinical aspects. Med Mycol. 2015;53(1):15–21. 10.1093/mmy/myu061 . 25477076

[15] Sanchotene KO, Madrid IM, Klafke GB, Bergamashi M, Terra PPD, Rodrigues AM, et al *Sporothrix brasiliensis* outbreaks and the rapid emergence of feline sporotrichosis. Mycoses. 2015;58(11):652–8. 10.1111/myc.12414 26404561

[16] Rodrigues AM, de Hoog S, de Camargo ZP. Emergence of pathogenicity in the *Sporothrix schenckii* complex. Med Mycol. 2013;51(4):405–12. 10.3109/13693786.2012.719648 . 22989196

[17] Coles FB, Schuchat A, Hibbs JR, Kondracki SF, Salkin IF, Dixon DM, et al A multistate outbreak of sporotrichosis associated with *Sphagnum* moss. Am J Epidemiol. 1992;136(4):475–87. 1415167 10.1093/oxfordjournals.aje.a116521

[18] Teixeira MM, de Almeida LG, Kubitschek-Barreira P, Alves FL, Kioshima ES, Abadio AK, et al Comparative genomics of the major fungal agents of human and animal Sporotrichosis: *Sporothrix schenckii* and *Sporothrix brasiliensis* . BMC Genomics. 2014;15:943 Epub 2014/10/30. 10.1186/1471-2164-15-943 . 25351875 PMC4226871

[19] Arrillaga-Moncrieff I, Capilla J, Mayayo E, Marimon R, Mariné M, Gené J, et al Different virulence levels of the species of *Sporothrix* in a murine model. Clin Microbiol Infect. 2009;15(7):651–5. 10.1111/j.1469-0691.2009.02824.x 19624508

[20] Rodrigues AM, Kubitschek-Barreira PH, Fernandes GF, de Almeida SR, Lopes-Bezerra LM, de Camargo ZP. Immunoproteomic analysis reveals a convergent humoral response signature in the *Sporothrix schenckii* complex. J Proteomics. 2015;115:8–22. 10.1016/j.jprot.2014.11.013 . 25434489

[21] Song Y, Li SS, Zhong SX, Liu YY, Yao L, Huo SS. Report of 457 sporotrichosis cases from Jilin province, northeast China, a serious endemic region. J Eur Acad Dermatol Venereol. 2013;27(3):313–8. 10.1111/j.1468-3083.2011.04389.x 22176524

[22] Fisher MC, Henk DA, Briggs CJ, Brownstein JS, Madoff LC, McCraw SL, et al Emerging fungal threats to animal, plant and ecosystem health. Nature. 2012;484(7393):186–94. 10.1038/nature10947 22498624 PMC3821985

[23] Olson DH, Aanensen DM, Ronnenberg KL, Powell CI, Walker SF, Bielby J, et al Mapping the global emergence of *Batrachochytrium dendrobatidis*, the amphibian chytrid fungus. PLoS ONE. 2013;8(2):e56802 10.1371/journal.pone.0056802 23463502 PMC3584086

[24] Lorch JM, Meteyer CU, Behr MJ, Boyles JG, Cryan PM, Hicks AC, et al Experimental infection of bats with *Geomyces destructans* causes white-nose syndrome. Nature. 2011;480(7377):376–8. Epub 2011/10/28. 10.1038/nature10590 . 22031324

[25] Martel A, Blooi M, Adriaensen C, Van Rooij P, Beukema W, Fisher MC, et al Recent introduction of a chytrid fungus endangers Western Palearctic salamanders. Science. 2014;346(6209):630–1. 10.1126/science.1258268 25359973 PMC5769814

[26] Rodrigues AM, Fernandes GF, Araujo LM, Della Terra PP, Dos Santos PO, Pereira SA, et al Proteomics-based characterization of the humoral immune response in sporotrichosis: Toward discovery of potential diagnostic and vaccine antigens. PLoS Negl Trop Dis. 2015;9(8):e0004016 10.1371/journal.pntd.0004016 26305691 PMC4549111

[27] Beurmann L, Gougerot H. Les Sporotrichose. Paris: Librairie Felix Alcan; 1912.

[28] Helm M, Berman C. The clinical, therapeutic and epidemiological features of the sporotrichosis infection on the mines. Proceedings of the Transvaal Mine Medical Officers’ Association. Sporotrichosis infection on mines of the Witwatersrand. Johannesburg, South Africa. 1947. p. 59–67.

[29] Taylor LH, Latham SM, woolhouse MEJ. Risk factors for human disease emergence. Philos Trans R Soc Lond B Biol Sci. 2001;356(1411):983–9. 10.1098/rstb.2001.0888 11516376 PMC1088493

[30] Rodrigues AM, de Hoog GS, de Camargo ZP. Molecular diagnosis of pathogenic *Sporothrix* species. PLoS Negl Trop Dis. 2015;9(12):e0004190 10.1371/journal.pntd.0004190 26623643 PMC4666615

[31] Rodrigues AM, Najafzadeh MJ, de Hoog GS, de Camargo ZP. Rapid identification of emerging human-pathogenic *Sporothrix* species with rolling circle amplification. Front Microbiol. 2015;6:1385 10.3389/fmicb.2015.01385 26696992 PMC4672047

